# COL22A1 Activates the PI3K/AKT Signaling Pathway to Sustain the Malignancy of Glioblastoma

**DOI:** 10.1155/ijog/6587097

**Published:** 2025-05-21

**Authors:** Tao Zheng, Yuanzhi Huang, Dong Chu, Shiming He

**Affiliations:** Department of Neurosurgery, Xi'an International Medical Center Hospital, Xi'an City, Shaanxi Province, China

**Keywords:** collagens, GBM, invasion, migration, proliferation

## Abstract

**Background:** Glioblastoma (GBM) represents an aggressive malignancy in the central nervous system, with a poor prognosis. Despite ongoing research efforts, there is still a lack of effective treatments, leading to the need for new therapeutic targets. Collagen plays a crucial role in the extracellular matrix and can impact the progression of cancer. Yet the potential involvement of COL22A1 (Collagen Type XXII Alpha 1 chain) in GBM has not been investigated.

**Materials and Methods:** The expression of COL22A1 was evaluated in both clinical GBM samples and the Gene Expression Profiling Interactive Analysis (GEPIA) database. Following COL22A1 knockdown in GBM cells, functional assays were conducted to assess proliferation, migration, and invasion. The influence of COL22A1 on oncogenic signaling pathways was analyzed through luciferase reporter assays and interventions with pharmacological agents. In vivo experiments were performed using a nude mouse xenograft model.

**Results:** COL22A1 expression was significantly higher in GBM tissues and was linked with a poor prognosis. Silencing COL22A1 suppressed proliferation, migration, and invasion of GBM cells and impeded tumorigenesis in vivo. On a mechanistic level, COL22A1 impacted the PI3K/AKT signaling cascade, demonstrated by decreased FOXO transcriptional activity and lower levels of phosphorylated PI3K (p-PI3K) and phosphorylated AKT (p-AKT). Furthermore, stimulating the PI3K/AKT pathway partially mitigated the impact of COL22A1 silencing.

**Conclusion:** COL22A1 plays a crucial role in dictating the malignancy of GBM through regulating the PI3K/AKT signaling pathway. Targeting COL22A1 could present a novel approach for GBM management.

## 1. Introduction

Glioblastoma (GBM), arising from cells in the central nervous system, represents one of the most aggressive types of malignancies in the nervous system, accounting for 82% of malignant glioma cases [1]. This condition frequently results in severe neurological symptoms, which include limb weakness, visual and sensory disturbances, emotional and memory disorientation, irregularities in executive function, frequent headaches, and occasional seizures [2, 3]. Despite the emergence of new treatment options, the median survival time is still under 2.5 years, with a survival rate of only 3% [4]. Additionally, most GBM patients experience postoperative recurrence [2]. Deregulation of different signaling pathways has been implicated in GBM progression [5, 6]. Therefore, elucidating the molecular mechanisms underlying GBM malignancy offers novel targets for therapeutic intervention.

Collagens, as the primary proteins found in the extracellular matrix (ECM), play a vital role in providing structural support to tissues and organs. Mutations in collagen genes can cause specific genetic disorders or conditions that alter connective tissues, as seen in various types of Ehlers–Danlos syndrome or osteogenesis imperfecta [7, 8]. In cancer biology, collagen is an essential component of the ECM, playing a significant role in cancer [9]. Changes in collagen gene expression can greatly affect the structure and makeup of the ECM in the tumor microenvironment, influencing cancer cell behavior such as invasion and metastasis [10].

Additionally, abnormal collagen deposition and remodeling within the tumor tissues support cancer cell migration and colonization at distant sites, as well as the establishment of neovasculature in tumor progression [11–13]. COL22A1 (Collagen Type XXII Alpha 1 chain) is a member of the collagen family structurally aligned with the fibril-associated collagen with interrupted triple-helices (FACIT) protein family. Dysregulation of COL22A1 has been observed in various cancers, such as head and neck squamous cell carcinoma and breast cancer [14–16]. There is evidence suggesting the potential oncogenic role of COL22A1 in gliomas [17]. However, the biological functions of COL22A1 in GBM remain to be thoroughly investigated.

The phosphatidylinositol 3-kinase/protein kinase B (PI3K/AKT) signaling pathway plays a crucial role in the pathogenesis and progression of GBM [18]. This pathway is frequently dysregulated in GBM, contributing to enhanced cell proliferation, survival, and invasion. Recent studies have shown that PI3K/AKT signaling is involved in maintaining the stemness of glioma stem cells, which are believed to be responsible for tumor initiation, recurrence, and therapy resistance [19]. Moreover, the activation of this pathway has been associated with poor prognosis and reduced overall survival in GBM patients [20]. Targeting the PI3K/AKT pathway has emerged as a promising therapeutic strategy, with several inhibitors currently under investigation in clinical trials [21]. However, the complex network of feedback loops and crosstalk with other signaling pathways often leads to resistance to single-agent PI3K/AKT inhibitors, highlighting the need for combination therapies and identification of novel regulators of this pathway in GBM [22].

This study investigates COL22A1's role in GBM and its underlying mechanisms. We examined COL22A1 expression in GBM tissues, assessed the impact of its knockdown on GBM cell behavior through in vitro and in vivo experiments, and explored its effects on oncogenic signaling pathways. Our findings reveal COL22A1 as a potential prognostic marker and therapeutic target in GBM, highlighting its role in promoting tumor aggression via the PI3K/AKT pathway. These results advance our understanding of GBM biology and suggest new avenues for targeted therapies.

## 2. Methods

### 2.1. Patient Specimens

Surgically removed GBM tissues were obtained from 40 patients who were diagnosed with GBM pathologically and had not undergone chemotherapy or radiotherapy previously. Inclusion criteria for GBM patients were as follows: age 30–45 years, newly diagnosed primary GBM (WHO Grade IV), and Karnofsky Performance Status ≥ 70. Patients with secondary GBM or other concurrent malignancies were excluded. Control samples consisted of normal brain tissues from 10 patients who had brain tissue removed due to craniocerebral injury. Control subjects were selected based on the following: age 30–45 years, no history of neurological disorders or brain tumors, and tissue samples obtained from noneloquent areas of the brain during decompressive surgery. The study received approval from the Institutional Ethics Review Board at Xi'an International Medical Center Hospital (2020-075), and all participating patients provided informed consent.

### 2.2. Cell Lines and Transfection

LN229, A172, and U87 GBM cell lines (Pricella, Wuhan, China) and normal human astrocyte (NHA) cells (Lonza in Walkersville, MD, United States) were used in this study. LN229 and A172 were maintained in DMEM medium from Pricella, while U87 was cultured in MEM medium (Pricella). Both DMEM and MEM media were supplemented with 10% FBS and 1% penicillin/streptomycin (Pricella). NHA cells were grown in astrocyte medium from ScienCell in San Diego, MD, United States. The cells were cultivated in a humidified atmosphere of 37°C with 5% CO_2_. Short hairpin RNA (shRNA) targeting COL22A1 (sh-COL22A1#1 5⁣′-GAAACCCTGCGTCGGCTTATT-3⁣′ and sh-COL22A1#2 5⁣′-GGGAACCTGGCTATGCTAAAG-3⁣′) and the negative control (sh-NC, ATCGGTAGCTCCATTACGCGT) in pLKO.1-puro vector were procured from HonorGene (Beijing, China). Transfection was carried out using the Lipofectamine 2000 transfection kit (11668030, Invitrogen, Shanghai, China) following the provided instructions. After transfection, cells stably integrated with shRNA were selected by 1 *μ*g/mL puromycin for 14 days before further experiments.

### 2.3. RNA Isolation and Quantitative RT-PCR

Total RNA specimens were purified following the manufacturer's instructions using TRIzol Reagent (R0016, Beyotime, Beijing, China). The extracted RNA was then converted into cDNA using oligo-deoxythymidine primers with the PrimeScript RT Reagent Kit (RR037A, Takara, Tokyo, Japan). The cDNA was amplified and quantified using the SYBR Premix Ex Taq II kit (RR820A, Takara, Tokyo, Japan) on an ABI7500 qPCR instrument (Applied Biosystems, San Diego, CA, United States). Data analysis was performed using the 2^−ΔΔCt^ approach. The following primers were synthesized by Sangon Biotech (Shanghai, China): COL22A1 forward primer: CCTAGCGTTCGTGTAGAAGGA; COL22A1 reverse primer: CCCATCCGTACATAGGAACTCT; GAPDH forward primer: GCAAATTCCATGGCACCGT; GAPDH reverse primer: TCGCCCCACTTGATTTTGG.

### 2.4. Cell Counting Kit-8 (CCK-8) Assays

To measure the growth of GBM cells, we employed the CCK-8 assay kit (CK04, Dojindo Molecular Technologies, Kumamoto, Japan) following the instructions provided by the manufacturer. The GBM cells were grown in 96-well plates with an initial seeding density of 1000 cells per well. The CCK-8 reagent (10 *μ*g) was added to each well at specified time points (0, 24, 48, and 72 h). After a 2-h incubation, we quantified the proliferation of GBM cells using a microplate reader (318C-Microplate reader, China) at a wavelength of 450 nm.

### 2.5. Transwell Migration/Invasion Assay

Prior to conducting our experiments, a Transwell system was assembled utilizing an 8 *μ*m pore size polycarbonate (PC) membrane (Corning, COR3422, Corning, NY, United States), in accordance with the provided guidelines. In the case of the invasion assay, the upper chamber was precoated with Matrigel (356234, BD Biosciences, Franklin Lakes, NJ, United States) prior to cell introduction. GBM cells that had undergone transfection were placed in a serum-free medium, resulting in a cell density of 2 × 10^6^ cells/mL. Subsequently, 200 *μ*L of this cell suspension was introduced into the upper chamber, while the lower chamber was filled with 700 *μ*L of medium containing 10% FBS. The Transwell system was maintained at a temperature of 37°C within a 5% CO_2_ environment. Following a 24-h incubation period, the cells that had migrated or invaded were fixed using ice-cold methanol and stained with 0.2% crystal violet (ZY548, Zeye Biotech, Shanghai, China) before image analysis. For cell counting, ImageJ (version 1.53c, National Institutes of Health, Bethesda, MD, United States) was utilized. Images of five random fields for each sample were captured using a microscope. These images were opened in ImageJ and converted to 8-bit grayscale, and the threshold was adjusted (image > adjust > threshold) until cells were clearly distinguishable from the background. If cells were touching, they were separated using process > binary > watershed. The “Analyze Particles” function was employed to count the cells, with appropriate size ranges set to exclude debris and include only whole cells. “Summarize” and “Display results” options were checked. The total cell count displayed by ImageJ was recorded for each field. The average number of cells per field for each sample was calculated.

### 2.6. Immunoblotting

GBM cells at 70%–90% confluence were harvested and lysed using radioimmunoprecipitation assay (RIPA) buffer (89900, ThermoFisher Scientific, Waltham, MA, United States) supplemented with Halt Protease Inhibitor Cocktail (78430, ThermoFisher Scientific) and Halt Phosphatase Inhibitor Cocktail (78420, ThermoFisher Scientific). Protein concentration was determined using a BCA protein assay kit (Beyotime Biotechnology, Shanghai, China). Protein samples (20 *μ*g) were mixed with 4X Laemmli sample loading buffer (1610747, Bio-Rad, Hercules, CA, United States) (containing bromophenol blue as the tracking dye) and denatured at 95°C for 5 min. The samples were then separated on 10% SDS-PAGE gels (4561033, Bio-Rad). Proteins were transferred to PVDF membranes (1620177, Bio-Rad) using precut blotting sandwiches (1704270, Bio-Rad). Membranes were blocked with 0.5% skimmed milk at room temperature and then washed three times with TBST for 10 min each. Primary antibodies (anti-PI3K (ab180967, 1 *μ*g/mL), anti-p-PI3K (ab138364, 1 *μ*g/mL), anti-AKT1 (ab308381, 1 *μ*g/mL), anti-p-AKT1 (ab81283, 1 *μ*g/mL), and anti-GAPDH (ab8245, 0.5 *μ*g/mL), all from Abcam, Cambridge, United Kingdom) and anti-COL22A1 (ABIN2790745, 2 *μ*g/mL, http://antibodies-online.com, Limerick, PA, United States) were applied to label the membrane overnight, followed by incubation with appropriate HRP-conjugated secondary antibodies (antirabbit IgG, HRP-linked antibody (ab6721, 0.2 *μ*g/mL) and antimouse IgG, HRP-linked antibody (ab6728, 0.2 *μ*g/mL), both from Abcam, Cambridge, United Kingdom). Blotting signals were visualized using ChemiDoc XRS^+^ System with Image Lab Software (1708265, Bio-Rad) after chemiluminescence development (34580, SuperSignal West Pico PLUS Chemiluminescent Substrate, Thermo Fisher Scientific).

### 2.7. Cell Signaling Luciferase Assay

To evaluate the effect of COL22A1 on classical signaling pathways, we employed the Cignal reporter assays kit (336841, Qiagen, Hilden, Germany). Briefly, 1 × 10^5^ GBM cells were plated in 24-well plates and allowed for cell attachment overnight. Subsequently, cells with either sh-NC or sh-COL22A1 expression were transfected with luciferase reporters of different signaling pathways, respectively, using Lipofectamine 2000 kit. Following a 36-h incubation period, cellular lysates were harvested, and the levels of firefly/renilla luciferase were measured using the Dual-Luciferase Reporter Assay System (E1910, Promega Corporation, Madison, WI, United States) according to the supplier's protocols.

### 2.8. In Vivo Assays

Male nude mice at 5 weeks old (weighing 15–20 g) were procured from the Experimental Animal Center at Wuhan University in China. They were housed individually in cages following a 12-h light–dark cycle, with free access to food and water. In the in vivo trials, U87 cells expressing sh-COL22A1 or sh-NC (5 × 10^6^ cells) were implanted subcutaneously into the mice (*n* = 5 animals in each group). Tumor size was measured at 7-day intervals for a total of 5 weeks. After the study period, tumors were removed from the euthanized mice and weighed. The tumor samples were then collected for immunohistochemistry (IHC) examination using anti-Ki67 (#9449; Cell Signaling Technologies) and anti-COL22A1 antibody (ab121846, Abcam) as described previously [23]. To quantify the IHC staining intensity and extent, the *H*-score was calculated. The staining intensity was categorized as 0 (*negative*), 1 (*weak*), 2 (*moderate*), or 3 (*strong*), and the percentage of positively stained cells for each intensity level was determined. The *H*-score was then calculated using the formula: *H*‐score = *Σ* (*i* × *Pi*), where *i* represents the intensity score and *Pi* is the corresponding percentage of stained cells. The final *H*-score ranged from 0 to 300, with 300 indicating 100% of cells stained strongly positive (3+). This research protocol gained approved by the Institutional Animal Care and Ethics Committee at Xi'an International Medical Center Hospital (2023-DL120).

### 2.9. Statistical Analysis

Data analysis was carried out using GraphPad Prism version 8 software (GraphPad, La Jolla, CA, United States), and results were presented as the mean plus/minus the standard deviation. The statistical methods employed included one-way ANOVA (analysis of variance) and Student's *t*-test. A *p* value of less than 0.05 was set as the statistical significance threshold.

## 3. Results

### 3.1. Elevated COL22A1 Expression Is Associated With Dismal Prognosis in GBM Patients

**Figure 1:**
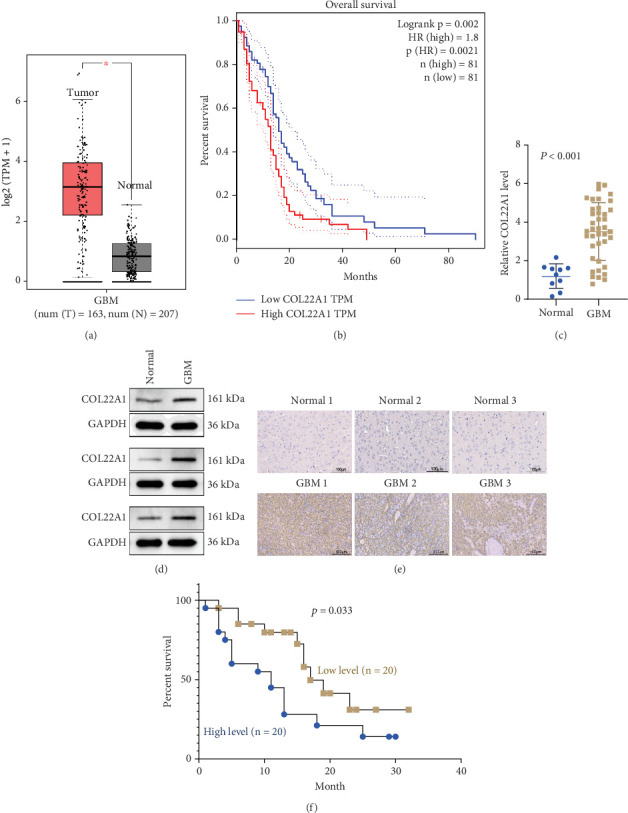
Elevated COL22A1 expression is associated with dismal prognosis in GBM patients. (a) COL22A1 expression levels in GBM and normal tissues from the GEPIA database. (b) Kaplan–Meier survival analysis of GBM patients based on COL22A1 expression from the GEPIA database. (c) RT-qPCR analysis of COL22A1 mRNA levels in GBM tissues (*n* = 40) and normal brain tissues (*n* = 10). (d) Western blot analysis of COL22A1 protein levels in GBM and normal brain tissues. (e) Immunohistochemistry staining of COL22A1 in t in GBM and normal brain tissues, scale bar: 100 *μ*m. (f) Kaplan–Meier survival analysis of 40 GBM patients in the high- and low-expression groups categorized by median COL22A1 mRNA expression value (median expression value is 1.395 and 3.59 in normal brain tissue and GBM samples, respectively).

According to the GBM cohort data in GEPIA database, COL22A1 expression levels were markedly higher in GBM tissues compared to noncancerous ones (Figure 1a). Patients diagnosed with GBM who exhibited increased expression of COL22A1 experienced a less favorable prognosis, as illustrated in Figure 1b. To further corroborate the observation in the database, analysis of COL22A1 levels was conducted using RT-qPCR in GBM tissue samples obtained during surgery (*n* = 40) and normal brain tissues obtained from individuals undergoing surgery for craniocerebral injury (*n* = 10). The mRNA expression levels of COL22A1 were significantly higher in GBM tissues compared to normal brain tissues, as depicted in Figure 1c. These results were further corroborated by western blot analysis (Figure 1d), as well as by the IHC staining of COL22A1 in tissue samples (Figure 1e). These findings demonstrate consistent upregulation of COL22A1 at both the mRNA and protein levels. Based on the median value of COL22A1 mRNA levels in GBM tissues, the 40 patients were categorized into high- and low-COL22A1 expression groups, with 20 patients showing high expression above the median value and 20 patients showing low expression below the median value. Survival analysis indicated a notably poorer clinical outcome in the high-COL22A1 group compared to the low-COL22A1 group, as shown in Figure 1f. In summary, elevated expression of COL22A1 was associated with an unfavorable prognosis in GBM patients.

### 3.2. COL22A1 Silencing Undermines the Malignant Features of GBM Cells

**Figure 2:**
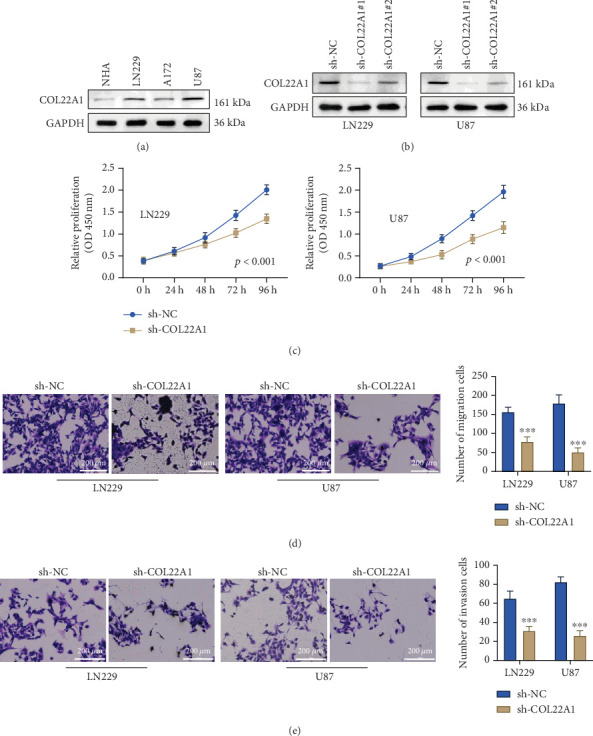
COL22A1 silencing undermines the malignant features of GBM cells. (a) Western blot analysis of COL22A1 expression in GBM cell lines (LN229, A172, and U87) and normal human astrocytes (NHAs). (b) Western blot confirming COL22A1 knockdown efficiency of sh-COL22A1#1 and #2 in LN229 and U87 cells. (c) CCK-8 assay showing cell proliferation in COL22A1-silenced and control LN229 and U87 cells. (d, e) Transwell assays demonstrating migration and invasion capabilities of COL22A1-silenced and control LN229 and U87 cells. Scale bar: 200 *μ*m.

In order to explore the engagement of COL22A1 in dictating aggressive characteristics of GBM cells, our study focused on the impact of silencing COL22A1 on their phenotypic traits. Initially, the levels of COL22A1 expression in GBM cells (LN229, A172, and U87) and NHA cells were assessed using western blot analysis. GBM cells displayed a notable increase in COL22A1 protein level compared to NHA cells (Figure 2a). Due to the prominent overexpression of COL22A1 in LN229 and U87 cells, these two GBM cell lines were chosen for further loss-of-function examinations. Stable GBM cell lines were generated using sh-NC, sh-COL22A1#1, and sh-COL22A1#2 transfection. Of the two shRNA constructs tested, sh-COL22A1#1 exhibited a more potent knockdown effect (Figure 2b) and was consequently chosen for subsequent experiments. Silencing of COL22A1 using this construct resulted in a significant reduction in the proliferative capacity of both LN229 and U87 cell lines (Figure 2c). Furthermore, the migratory and invasive capacities of both GBM cell lines were significantly impeded under the condition of COL22A1 knockdown (Figure 2d,e). Therefore, our results pinpoint COL22A1 as a critical factor to sustain the aggressive behaviors of GBM cells.

### 3.3. COL22A1 Silence Diminishes the Oncogenic PI3K/AKT Signaling Pathway

**Figure 3:**
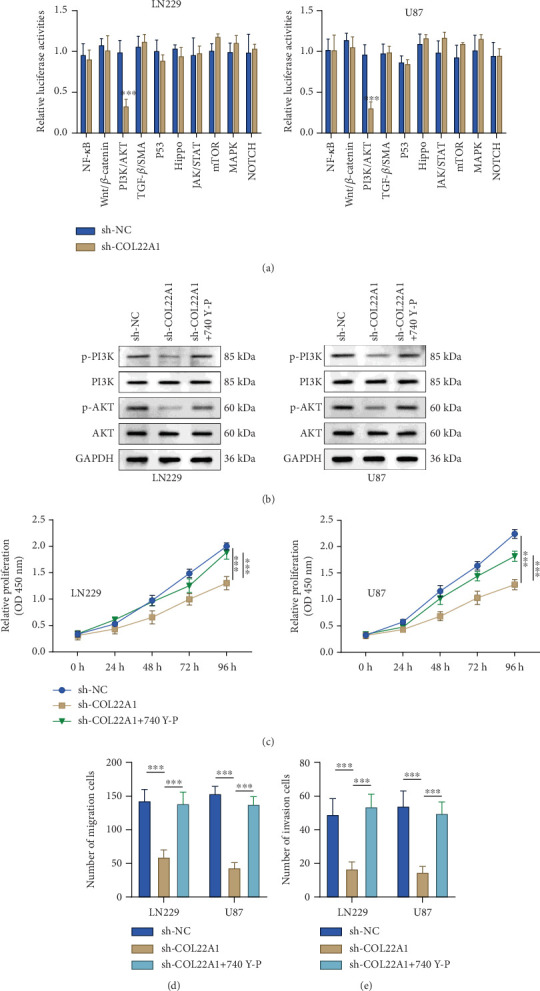
COL22A1 silence diminishes the oncogenic PI3K/AKT signaling pathway. (a) Luciferase reporter assays for different oncogenic signaling pathways in COL22A1-silenced (sh-COL22A1#1) and control (sh-NC) GBM cells. (b) Western blot analysis of p-PI3K and p-AKT levels in COL22A1-silenced GBM cells with or without 740 Y-P treatment. (c) CCK-8 assay showing cell proliferation in COL22A1-silenced GBM cells with or without 740 Y-P treatment. (d) Transwell migration assay and (e) invasion assay demonstrating migratory and invasive capabilities of COL22A1-silenced GBM cells with or without 740 Y-P treatment.

To uncover the signaling pathways involved in the biological function of COL22A1, we explored how COL22A1 silencing impacts traditional oncogenic signaling pathways. Notably, silencing COL22A1 (sh-COL22A1#1) specifically reduced the activity of the PI3K/AKT pathway. This was demonstrated by the decreased luciferase reporter activity of forkhead box O (FOXO) factors in both GBM cell lines (Figure 3a). Next, GBM cells expressing sh-COL22A1 were treated with 20 *μ*mol/L of 740 Y-P, a PI3K activator. Immunoblotting results confirmed that COL22A1 silencing lowered the levels of p-PI3K and p-AKT. However, treatment with 740 Y-P significantly reversed this effect (Figure 3b). Crucially, the decreased proliferation in U87 and LN229 cells after COL22A1 silencing was partly restored upon the introduction of 740 Y-P (Figure 3c). Furthermore, the inhibited migration and invasion of U87 and LN229 cells upon COL22A1 silencing were also partially rescued by 740 Y-P (Figure 3d,e). Collectively, our results imply that COL22A1 is required to support the malignant behavior of GBM cells via the PI3K/AKT signaling pathway.

### 3.4. COL22A1 Silencing Hinders Tumor Growth of GBM Cells In Vivo

**Figure 4:**
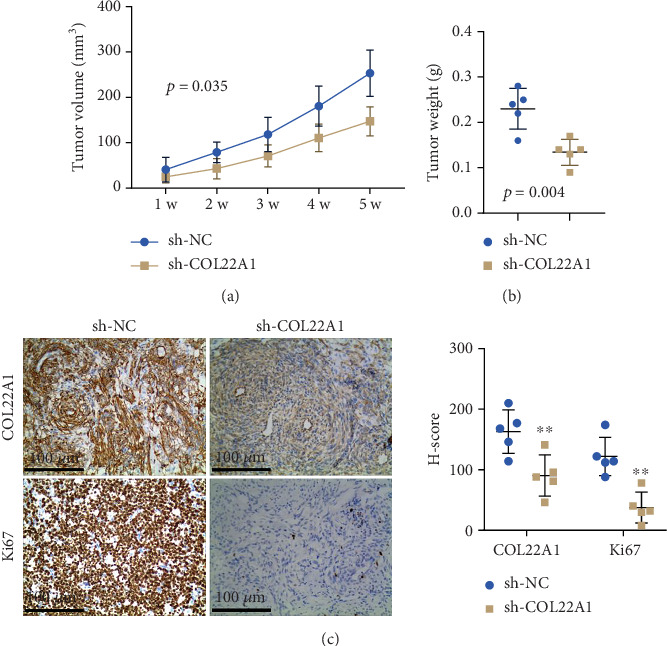
COL22A1 silencing hinders tumor growth of GBM cells in vivo. (a) Tumor volume growth curves of xenografts derived from COL22A1-silenced (sh-COL22A1) and control (sh-NC) U87 cells. (b) Tumor weights at the end of the xenograft experiment. (c) Immunohistochemical staining of Ki67 and COL22A1 in xenograft tumors derived from COL22A1-silenced and control GBM cells. *N* = 5 animals in each group. Scale bar: 100 *μ*M.

To investigate the effect of COL22A1 silencing in vivo, U87 cells expressing sh-COL22A1 or sh-NC were inoculated into nude mice to establish xenograft models. Notably, tumors expressing sh-COL22A1 exhibited significantly reduced volume growth compared to the sh-NC control group (Figure 4a). Furthermore, a marked decrease in tumor weight was observed in the COL22A1-silenced group (Figure 4b). IHC analyses further revealed reduced Ki67 expression in tumors in which COL22A1 was knocked down (Figure 4c). Together, these findings strongly suggest that COL22A1 is required to support rapid tumorigenesis of GBM cells.

## 4. Discussion

**Figure 5:**
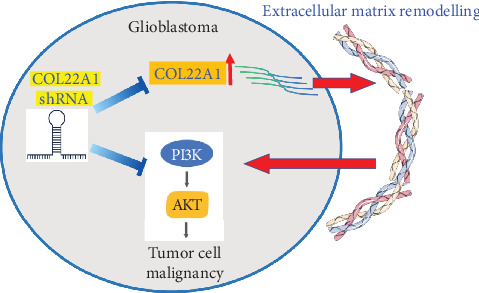
Mechanistic illustration of COL22A1 overexpression in GBM and the activation of the PI3K/AKT signaling pathway. COL22A1 upregulation in GBM activates the PI3K/AKT signaling pathway to sustain the malignancy of GBM cells.

In our current study, we have unveiled the functional engagement of COL22A1 in GBM progression and deciphered its underlying signaling pathway (Figure 5). High levels of COL22A1 were strongly associated with poor prognosis. Functionally, silencing COL22A1 in GBM cell lines (LN229 and U87) resulted in a marked reduction in their proliferation and hindered their ability to migrate and invade. In a mouse xenograft model, COL22A1 suppression impeded tumor formation of GBM cells, which was confirmed by reduced Ki67 expression. Moreover, COL22A1 was found to activate the PI3K/AKT pathway, evidenced by decreased FOXO transcriptional activity and diminished levels of phosphorylated PI3K and AKT upon silencing. Our findings indicate an indispensable role of COL22A1 in the malignant features of GBM cells, offering a novel therapeutic target for GBM treatment.

Recent evidence has pinpointed the potential role of COL22A1 in the tumorigenesis of gliomas and lung adenocarcinoma [17, 24]. As a scaffold protein involved in assembling the ECM, COL22A1, similar to other collagen genes, can impact various malignant characteristics in tumor cells [25, 26]. These include proliferation, migration, invasion, and angiogenesis. COL22A1 has been previously demonstrated to promote the survival of nasopharyngeal carcinoma cells by abrogating cellular senescence [27]. Additionally, in the lung adenocarcinoma cohort from The Cancer Genome Atlas (TCGA) database, COL22A1 has been identified as a key predictor of prognosis in a well-crafted multivariate risk model [24]. COL22A1 overexpression is linked to lymph node metastasis, pathological stage, and a poor patient outcome in the case of head and neck cancer [15]. Furthermore, it serves as a unique signature of immune profiles in osteosarcoma and represents a crucial prognostic factor [28].

Our findings on COL22A1 in GBM align with and extend previous observations in other cancer types, supporting a conserved oncogenic role for this ECM protein. By demonstrating COL22A1's impact on GBM cell behavior through the PI3K/AKT pathway, this study provides novel mechanistic insights into how COL221A influence intracellular signaling to promote tumor progression. The consistent association of COL22A1 with poor prognosis across various cancers, including GBM, reinforces its potential as both a prognostic biomarker and a therapeutic target. Future investigations into COL22A1's role in cellular senescence and immune modulation in GBM could further elucidate its multifaceted functions in cancer biology, building on existing knowledge from other tumor types.

Tumorigenesis and metastasis during cancer development inherently involve the hyperactivation of classic oncogenic signaling pathways, among which the Wnt/*β*-catenin, Notch, and PI3K/AKT signaling pathways are widely acknowledged [11, 29–31]. The dysregulation of oncogenes and tumor suppressors intricately shapes these signaling cascades, ultimately dictating the fate of tumor cells. In our research, we methodically analyzed various oncogenic pathways and identified the PI3K/AKT pathway as the primary target influenced by COL22A1. Considerable evidence highlights the crucial function of the PI3K/AKT signaling pathway in the initiation and advancement of GBM [32–34]. Our results demonstrated that pharmaceutical activation of the PI3K/AKT signaling pathway could counteract the suppressive effects of COL22A1 gene silencing on the aggressive behavior of GBM cells, further highlighting the crucial role of this oncogenic pathway in sustaining GBM malignancy.

Our findings on COL22A1's role in GBM malignancy align with the growing evidence highlighting the importance of ECM components in GBM biology. The association between COL22A1 expression and poor prognosis is consistent with the observation that collagen architecture influences GBM patient survival [35]. Furthermore, Tsai et al.'s work on Collagen V Alpha 1 chain suggests that multiple collagen types contribute to GBM malignancy through ECM remodeling [36]. In line with this, it has been proposed to leverage the ECM to enhance immunotherapy efficacy in GBM [37]. Furthermore, the activation of PI3K/AKT signaling by COL22A1 in our study complements the findings of Langhans et al., in which the authors demonstrated that PI3K signaling inhibitors curtail the aggressiveness of GBM cells [38]. There is also evidence that protein arginine methyltransferase 5 (PRMT5) activates the PI3K/AKT signaling pathway to support tumor metastasis [39]. However, whether this pathway is related to the activation of PI3K/AKT signaling in GBM remains to be clarified. We postulate that advances in targeting the PI3K/AKT signaling pathway could significantly improve survival outcomes for GBM patients [40, 41].

While our study provides valuable insights into the role of COL22A1 in GBM progression, it has some limitations. We focused primarily on the PI3K/AKT pathway, but did not explore potential interactions with other key regulators like phosphatase and tensin homolog (PTEN), a major tumor suppressor frequently mutated in GBM [42]. Future studies should investigate how COL22A1 might interact with or influence PTEN activity, as PTEN is a critical negative regulator of the PI3K/AKT pathway [43]. Exploring combination therapies targeting both COL22A1 and PTEN could potentially enhance treatment efficacy. Additionally, the biological function of COL22A1 should be further validated using gene knockout approach and orthotopic animal models. Further research is also needed to elucidate the precise mechanisms by which COL22A1 activates PI3K/AKT signaling and to determine if COL22A1 inhibition could sensitize GBM cells to existing therapies. Moreover, other signaling pathways such as MAPK/ERK, JAK/STAT, and Wnt/*β*-catenin should also be explored to gain a more comprehensive understanding of COL22A1's role in GBM pathogenesis, as these pathways are known to be implicated in GBM progression and may interact with or be influenced by COL22A1 activity.

In summary, this study identifies COL22A1 as a key promoter of GBM progression through activation of the PI3K/AKT pathway. Elevated COL22A1 expression correlates with poor prognosis and is required to sustain the malignancy of GBM cells. These findings highlight COL22A1 as a potential prognostic biomarker and therapeutic target in GBM. Future research should focus on elucidating the precise mechanisms of COL22A1-mediated PI3K/AKT activation, exploring its therapeutic potential, and investigating its role in treatment resistance and tumor microenvironment modulation in GBM.

## Data Availability

The datasets used and/or analyzed during the current study are available from the corresponding author via email request.

## References

[1] Mckinnon C., Nandhabalan M., Murray S. A., Plaha P. (2021). Glioblastoma: Clinical Presentation, Diagnosis, and Management. *BMJ*.

[2] Minniti G., Niyazi M., Alongi F., Navarria P., Belka C. (2021). Current Status and Recent Advances in Reirradiation of Glioblastoma. *Radiation Oncology*.

[3] Luo C., Song K., Wu S. (2021). The Prognosis of Glioblastoma: A Large, Multifactorial Study. *British Journal of Neurosurgery*.

[4] Le Rhun E., Preusser M., Roth P. (2019). Molecular Targeted Therapy of Glioblastoma. *Cancer Treatment Reviews*.

[5] Khabibov M., Garifullin A., Boumber Y. (2022). Signaling Pathways and Therapeutic Approaches in Glioblastoma Multiforme (Review). *International Journal of Oncology*.

[6] Orozco-Mera J., Peralta-Pizza F., Escobar-Vidarte O., Coll-Tello B., Ariza A. (2023). Signaling Pathways in the Relapse of Glioblastoma. *Oncology (Williston Park)*.

[7] Campos L. D., Santos Junior V. A., Pimentel J. D., Carregã G. L. F., Cazarin C. B. B. (2023). Collagen Supplementation in Skin and Orthopedic Diseases: A Review of the Literature. *Heliyon*.

[8] Jovanovic M., Guterman-Ram G., Marini J. C. (2022). Osteogenesis Imperfecta: Mechanisms and Signaling Pathways Connecting Classical and Rare OI Types. *Endocrine Reviews*.

[9] De Martino D., Bravo-Cordero J. J. (2023). Collagens in Cancer: Structural Regulators and Guardians of Cancer Progression. *Cancer Research*.

[10] Zhang Q., An Z. Y., Jiang W., Jin W. L., He X. Y. (2023). Collagen Code in Tumor Microenvironment: Functions, Molecular Mechanisms, and Therapeutic Implications. *Biomedicine & Pharmacotherapy*.

[11] Rømer A. M. A., Thorseth M. L., Madsen D. H. (2021). Immune Modulatory Properties of Collagen in Cancer. *Frontiers in Immunology*.

[12] Patwardhan S., Mahadik P., Shetty O., Sen S. (2021). ECM Stiffness-Tuned Exosomes Drive Breast Cancer Motility Through Thrombospondin-1. *Biomaterials*.

[13] Han X., Caron J. M., Brooks P. C. (2020). Cryptic Collagen Elements as Signaling Hubs in the Regulation of Tumor Growth and Metastasis. *Journal of Cellular Physiology*.

[14] Wu Z. H., Li C., Zhang Y. J., Zhou W. (2022). Identification of a Cancer Stem Cells Signature of Head and Neck Squamous Cell Carcinoma. *Frontiers in Genetics*.

[15] Misawa K., Kanazawa T., Imai A. (2014). Prognostic Value of Type XXII and XXIV Collagen mRNA Expression in Head and Neck Cancer Patients. *Molecular and Clinical Oncology*.

[16] Ademuyiwa F. O., Chen I., Luo J. (2021). Immunogenomic Profiling and Pathological Response Results From a Clinical Trial of Docetaxel and Carboplatin in Triple-Negative Breast Cancer. *Breast Cancer Research and Treatment*.

[17] Liu H., Zeng Z., Sun P. (2023). Prognosis and Immunoinfiltration Analysis of Angiogene-Related Genes in Grade 4 Diffuse Gliomas. *Aging (Albany NY)*.

[18] Zając A., Sumorek-Wiadro J., Langner E. (2021). Involvement of PI3K Pathway in Glioma Cell Resistance to Temozolomide Treatment. *International Journal of Molecular Sciences*.

[19] Eckerdt F. D., Bell J. B., Gonzalez C. (2020). Combined PI3K*α*-mTOR Targeting of Glioma Stem Cells. *Scientific Reports*.

[20] da Silva A. L. L., de Araújo T. P. G., de Albuquerque Ferreira S. C. (2024). PI3K Signaling Pathways as a Molecular Target for Glioblastoma Multiforme. *Current Protein & Peptide Science*.

[21] Narayan R. S., Fedrigo C. A., Brands E. (2017). The Allosteric AKT Inhibitor MK2206 Shows a Synergistic Interaction With Chemotherapy and Radiotherapy in Glioblastoma Spheroid Cultures. *BMC Cancer*.

[22] Mishra V. S., Kumar N., Raza M., Sehrawat S. (2020). Amalgamation of PI3K and EZH2 Blockade Synergistically Regulates Invasion and Angiogenesis: Combination Therapy for Glioblastoma Multiforme. *Oncotarget*.

[23] Wu J., Liu Z., Huang T. (2023). Cerebrospinal Fluid Circulating Tumor DNA Depicts Profiling of Brain Metastasis in NSCLC. *Molecular Oncology*.

[24] Dong L., Fu L., Zhu T. (2023). A Five-Collagen-Based Risk Model in Lung Adenocarcinoma: Prognostic Significance and Immune Landscape. *Frontiers in Oncology*.

[25] Shi R., Zhang Z., Zhu A. (2022). Targeting Type I Collagen for Cancer Treatment. *International Journal of Cancer*.

[26] Angre T., Kumar A., Singh A. K., Thareja S., Kumar P. (2022). Role of Collagen Regulators in Cancer Treatment: A Comprehensive Review. *Anti-Cancer Agents in Medicinal Chemistry*.

[27] Huang M. L., Luo W. L. (2022). Engrailed Homeobox 1 Transcriptional Regulation of COL22A1 Inhibits Nasopharyngeal Carcinoma Cell Senescence Through the G1/S Phase Arrest. *Journal of Cellular and Molecular Medicine*.

[28] Pan R., Pan F., Zeng Z. (2022). A Novel Immune Cell Signature for Predicting Osteosarcoma Prognosis and Guiding Therapy. *Frontiers in Immunology*.

[29] Zhou B., Lin W., Long Y. (2022). Notch Signaling Pathway: Architecture, Disease, and Therapeutics. *Signal Transduction and Targeted Therapy*.

[30] Yu F., Yu C., Li F. (2021). Wnt/*β*-Catenin Signaling in Cancers and Targeted Therapies. *Signal Transduction and Targeted Therapy*.

[31] Su W. Y., Tian L. Y., Guo L. P., Huang L. Q., Gao W. Y. (2023). PI3K Signaling-Regulated Metabolic Reprogramming: From Mechanism to Application. *Biochimica et Biophysica Acta (BBA)-Reviews on Cancer*.

[32] Mohamed E., Kumar A., Zhang Y. (2022). PI3K/AKT/mTOR Signaling Pathway Activity in IDH-Mutant Diffuse Glioma and Clinical Implications. *Neuro-Oncology*.

[33] Daisy Precilla S., Biswas I., Kuduvalli S. S., Anitha T. S. (2022). Crosstalk Between PI3K/AKT/mTOR and WNT/*β*-Catenin Signaling in GBM - Could Combination Therapy Checkmate the Collusion?. *Cellular Signalling*.

[34] Barzegar Behrooz A., Talaie Z., Jusheghani F., Łos M. J., Klonisch T., Ghavami S. (2022). Wnt and PI3K/Akt/mTOR Survival Pathways as Therapeutic Targets in Glioblastoma. *International Journal of Molecular Sciences*.

[35] Pointer K. B., Clark P. A., Schroeder A. B., Salamat M. S., Eliceiri K. W., Kuo J. S. (2017). Association of Collagen Architecture With Glioblastoma Patient Survival. *Journal of Neurosurgery*.

[36] Tsai H. F., Chang Y. C., Li C. H. (2021). Type V Collagen Alpha 1 Chain Promotes the Malignancy of Glioblastoma Through PPRC1-ESM1 Axis Activation and Extracellular Matrix Remodeling. *Cell Death Discovery*.

[37] Collado J., Boland L., Ahrendsen J. T., Miska J., Lee-Chang C. (2024). Understanding the Glioblastoma Tumor Microenvironment: Leveraging the Extracellular Matrix to Increase Immunotherapy Efficacy. *Frontiers in Immunology*.

[38] Langhans J., Schneele L., Trenkler N. (2017). The Effects of PI3K-Mediated Signalling on Glioblastoma Cell Behaviour. *Oncogene*.

[39] Huang L., Zhang X. O., Rozen E. J. (2022). PRMT5 Activates AKT via Methylation to Promote Tumor Metastasis. *Nature Communications*.

[40] Colardo M., Segatto M., Di Bartolomeo S. (2021). Targeting RTK-PI3K-mTOR Axis in Gliomas: An Update. *International Journal of Molecular Sciences*.

[41] Hashemi M., Etemad S., Rezaei S. (2023). Progress in Targeting PTEN/PI3K/Akt Axis in Glioblastoma Therapy: Revisiting Molecular Interactions. *Biomedicine & Pharmacotherapy*.

[42] Li Y., Liang Y., Sun Z. (2019). Radiogenomic Analysis of PTEN Mutation in Glioblastoma Using Preoperative Multi-Parametric Magnetic Resonance Imaging. *Neuroradiology*.

[43] Claridge S. E., Hopkins B. D. (2022). Circling Back to PTEN: Fumarate Inhibits Canonical Tumor Suppressor. *Molecular Cell*.

